# Current Perspectives and Future Directions in Lung Transplantation

**DOI:** 10.3390/life13071432

**Published:** 2023-06-23

**Authors:** Alessandra Verzelloni Sef, Davorin Sef, Vladimir Trkulja, Nandor Marczin

**Affiliations:** 1 Department of Anaesthesia and Critical Care, Harefield Hospital, Royal Brompton and Harefield Hospitals, London UB9 6JH, UK; n.marczin@imperial.ac.uk; 2 Harefield Hospital, Royal Brompton and Harefield Hospitals, London UB9 6JH, UK; davorin.sef@gmail.com; 3 School of Medicine, University of Zagreb, 10000 Zagreb, Croatia; vladimir.trkulja@mef.hr; 4 Division of Anaesthetics, Pain Medicine and Intensive Care, Department of Surgery and Cancer, Imperial College London, London SW7 2BX, UK

This Special Issue of Life features compelling original research and reviews related to current trends in lung transplantation (LTx). These articles encompass challenges and questions across the LTx field, providing useful insights into perioperative management, intraoperative circulatory support, postoperative rehabilitation, and follow-up of LTx recipients. The field of LTx has emerged as a multidisciplinary specialty, and members of the team include cardiothoracic and transplant surgeons, anaesthetists, intensivists, respiratory physicians, physiotherapists, and pathologists. Patients with end-stage lung failure undergoing LTx present a few unique challenges. However, continuous advancements and achievements over the last several decades have contributed to improved outcomes of these high-risk patients [1,2,3,4,5]. The use of cardiopulmonary bypass (CPB) was necessary in the early stages of LTx development. However, over the last decade, the utilization of CPB has decreased due to advances in surgical technique and the established utilization of intraoperative extracorporeal membrane oxygenation (ECMO) [5]. Furthermore, minimally invasive LTx via bilateral anterior thoracotomies has emerged simultaneously as a superior surgical strategy with early postoperative and mid-term clinical benefits compared with the traditional surgical approach [6,7].

The importance and choice of intraoperative mechanical circulatory support (MCS) in LTx is highlighted by Starke H. and colleagues [8] who summarised the current trends and evidence for intraoperative MCS in their review article. In particular, the authors emphasized the value of the ERSAS (early risk stratification and strategy) concept that can be helpful in identifying potential risk factors and developing an appropriate therapeutic strategy for the optimal utilization of MCS in order to reduce the risk of complications [4,8]. Based on recent evidence, when indicated, venoarterial (VA) ECMO is the preferable approach with superior postoperative outcomes compared to CPB [5,9]. Similarly, veno-venous (VV) ECMO as a bridge to transplant is a reasonable strategy for critically ill recipients that can be applied with acceptable operative mortality risk and 1-year survival that is comparable to non-bridged recipients [4]. Furthermore, ECMO support may provide the advantage of weaning patients off positive pressure mechanical ventilation and engaging in physical therapy in the immediate postoperative period. Importantly, the American Association for Thoracic Surgery Clinical Practice Standards Committee has recently developed an expert consensus and recommendations about the use of MCS before, during, and after LTx [10]. Importantly, recent consensus-based recommendations for anaesthetic and intensive care management in LTx redefined and emphasized the role of the anaesthesiologist and intensivist as integral and pivotal members of the multidisciplinary team and in decision-making [3]. Finally, the authors highlighted the challenges in the management of typical haemodynamic complications such as right ventricular failure, diastolic dysfunction caused by left ventricular deconditioning, and reperfusion injury to the transplanted lung [8].

In their narrative overview, Fessler and colleagues summarized the most recent data and knowledge in the anaesthetic management of LTx [11]. The authors described several important concerns, including the impact of coronavirus disease 2019 (COVID-19), future of LTx for cystic fibrosis (CF) patients, haemostasis management, expanding role of ECMO in LTx, early prediction of primary graft dysfunction, and pain management [11]. Recently, we have witnessed a reduction in LTx activity with up to 47% of centres limited LTx to only urgent cases, and the mortality on the waiting list has even increased up to 20%. Of particular interest is the report of 39,485 patients hospitalized for COVID-19 in Austria, of which 106 patients with COVID-19-related acute respiratory distress syndrome were referred to assess the necessity for LTx, and 19 (18%) underwent LTx [12].

In recent years, management of patients with CF has changed considerably since the introduction of CF transmembrane conductance regulator (CFTR) modulator therapies (elexacaftor–tezacaftor–ivacaftor) which can be recommended to all CF patients with advanced pulmonary disease and a Phe508del mutation before considering listing for LTx [13]. Furthermore, the authors reiterated that preoperative ECMO as a bridge to LTx represents a valuable support as an alternative to invasive mechanical ventilation due to the fact that awake ECMO can allow early ambulation, reduce in ventilator-associated pneumonia, and prevent skeletal muscle deconditioning.

In addition, while several studies reported an association between intraoperative transfusion of blood products and worse post-transplant outcomes, it has been highlighted that a point-of-care targeted coagulopathy management strategy may help in tailoring blood transfusions to the patient’s needs and improving outcomes. This is of paramount importance as increased intraoperative fluid volume has been associated with the development of the most severe form of primary graft dysfunction after LTx surgery [14,15,16,17].

Regarding pain management, thoracic epidural analgesia remains the gold standard, although some centres prefer avoiding this approach because of the risk of epidural haematoma and bleeding in case of potential use of MCS requiring heparinisation. In such cases, chest wall blocks are preferred, though they are often less effective.

The impact of structured physical training and pulmonary rehabilitation on exercise ability and quality of life (QoL) in LTx recipients became increasingly important in the last decade. Abidi and colleagues reviewed studies that investigated the impact of exercise training programmes on the QoL of LTx recipients before and after the transplantation [18]. LTx candidates are particularly characterised by limited training capacity and low average QoL. The six-minute walking test is still a crucial tool to assess the successful outcomes of the rehabilitation programme. Current data suggested that preoperative interval training is associated with the intensity of dyspnoea during exercise and can result in clinically relevant improvements in 6-minute walking distance and physical capacity in the early post-transplant period [18,19]. On the other hand, post-transplant rehabilitation resulted in significant improvements in FEV1 and FVC [20]. However, more prospective studies and large, well-designed, randomized controlled trials are required to determine the best exercise training and rehabilitation settings and their immediate and long-term impact on important post-LTx clinical outcomes, including time to discharge, rejection, infection, re-hospitalization, QoL, and survival.
